# Improving primary palliative care in Scotland: lessons from a mixed methods study

**DOI:** 10.1186/s12875-015-0391-x

**Published:** 2015-12-10

**Authors:** Bruce Mason, Susan Buckingham, Anne Finucane, Peter Hutchison, Marilyn Kendall, Hazel McCutcheon, Lorna Porteous, Scott A. Murray

**Affiliations:** The University of Edinburgh, Centre for Population Health Sciences, The Usher Institute of Population Health Sciences and Informatics, Primary Palliative Care Research Group, Medical School, Teviot Place, Edinburgh, EH8 9AG UK; Marie Curie Hospice Edinburgh, Edinburgh, UK; NHS Dumfries and Galloway, Dumfries, Scotland; NHS Lothian, Edinburgh, UK

**Keywords:** Primary healthcare, General practice, Palliative care, Qualitative research, End of life care, Terminal care

## Abstract

**Background:**

Since 2012, all GP practices across Scotland have been supported to take a systematic approach to end-of-life care, by helping them to identify more patients for palliative care through a Palliative Care Directed Enhanced Service (DES). We aimed to understand the impact of this initiative.

**Methods:**

Routine quantitative data from the 2012/13, and 2013/14 DES were collected from regional health boards, analysed and discussed. Qualitative data were collected from a sample of 2012/13 DES returns and analysed using Thematic Analysis.

**Results:**

Data were received from 512 practices in nine Scottish Health boards for the 2012-13 DES and 638 practices in 11 Health boards for 2013-14. A sample of 90 of the returns for 2012-13 was selected for qualitative analysis.

In 2012-13, 72 % of patients who died of cancer were listed on the palliative care register (PCR) before death while 27 % of patients who died as a result of non-malignant conditions were listed on the PCR. In 2013-14, cancer identification remained the same but identification of people dying with other long-term conditions had improved to 32.5 %.

We identified several key issues needed to improve palliative care in the community. The need for training to identify patients with palliative care needs (particularly non-cancer); communication skills training; improvements in sharing information across the NHS; under-resource of and lack of coordination with district nurses; improvements in information technology; and tools for working with enlarged palliative care registers.

**Conclusions:**

The DES helped more patients with long-term conditions (LTC) receive generalist palliative care. Approaching generalist palliative care as anticipatory care could facilitate communication between GPs and patients/families and remove some barriers to early identification of palliative care needs. Improvement of information technology and use of identification tools like the SPICT™ may improve professionals’ communication with each other and help may make identification and management of patients easier.

**Electronic supplementary material:**

The online version of this article (doi:10.1186/s12875-015-0391-x) contains supplementary material, which is available to authorized users.

## Background

An estimated 75 % of people who die need palliative care [1, 2]. Being identified for palliative care provided in the community by primary care teams (PCTs) leads to an increased likelihood that the recipient’s wishes around place of care and treatment objectives will be met, and that the recipient is likely to have fewer emergency admissions and spend less time in hospital during their last year of life [1, 3]. Research in 2012 [4] found that many patients were not being identified for inclusion on the GP palliative care register before death, or if they were identified they were identified very late in the illness trajectory [5]. Furthermore, those who were identified were overwhelmingly likely to suffer from cancer leading to an inequity of treatment for patients with other life-threatening long-term conditions (LTCs) [6].

In keeping with the Scottish End-of-Life Care plan 2008 and beyond [7, 8], the majority of GP practices across Scotland have taken part in a Palliative Care Directed Enhanced Service (DES) that started in 2012 [9]. This has been the main development in palliative care funding in Scotland recently. It aimed to support primary care teams (PCTs) to take a systematic approach to end-of-life care, by helping them to identify more patients for the palliative care register and to create electronic palliative care summaries (ePCS): a form of electronic record shared across NHS services with patient consent [10].

This paper reports an evaluation of the first two years of the DES. It provides an insight into the impact of the DES and the practical issues faced by GPs in attempting to deliver high-quality palliative care in the community.

## Methods

This was a mixed methods project combining qualitative and quantitative data. In this paper, we are reporting on routine quantitative data from the 2012/13, and 2013/14 DES, and qualitative data collected from a sample of DES returns from 2012-13: the 2013/14 qualitative information was not available during the project’s lifespan. The DES returns were completed by a practice manager or GP and sent to the local health board. Consequently we collected the returns from health boards who responded to our requests for the data.

Quantitative data was imported into SPSS version 21 and analysed using descriptive statistics. Qualitative data was anonymised and then imported into Word or PDF. The resulting qualitative data set underwent a qualitative content analysis [11, 12]. The results of the qualitative and quantitative analyses were then discussed by the co-authors as a multi-disciplinary steering group, in order to develop greater understanding of the best ways to identify and support people facing the end of life and to develop recommendations for good practice. All data was collected by the project manager, SB. Statistical analysis was performed by AF (Phd) and qualitative data was analysed by the lead researcher, BM (PhD). The lead researcher, BM, was a full-time research associate at the University of Edinburgh during the project with over 15 years experience in qualitative research methods.

### Ethics

Permission from South-east Scotland Ethics Committee to precede with this study as a service evaluation (NR/1402AB25) under the terms of the Governance Arrangements for Research Ethics Committees (A Harmonised Edition) was granted. Ethical approval from the University of Edinburgh Ethics Review Group was also granted. The Caldicott guardian judged that the planned use and handling of data was appropriate and indicated. All participants who took part in interviews or observed meetings gave written consent to participate.

## Results

Nine (of 12) health boards responded to our requests for the 2012-13 DES, allowing us to collect data from 512 practices equating to just over half of all general practices in Scotland. Eleven (of 12) health boards returned data for the 2013-14 DES, allowing us to collect returns from 638 practices. In both years, a proportion of the returns were excluded from the quantitative analysis due to missing, incomplete or erroneous data entries. We excluded 82 returns from 2012-13 DES (16 %) and 83 returns from 2013-14 (13 %). See Additional file 1 for an explanation of the exclusion criteria. Qualitative data from the 2012-13 return was extracted from a random sampling of 94 of the 512 returns received with a minimum of 6 per health board. Of these, 4 were excluded because the qualitative data was either missing in total (*n* = 3) or unintelligible (*n* = 1). This ensured that the spread of samples reflected the population areas of Scotland while ensuring that every health board (that returned data) would have a minimum number of returns analysed. Note that the random sampling did include all returns, even those with errors in their numerical returns (Table 1).

**Table 1:** Returns received and included

	Health boards returned	Total practices sampled	Returns excluded from quantitative analysis	Returns included in quantitative analysis	Returns included in qualitative analysis
DES 2012-13	9	512	82	430	90
DES 2013-14	11	638	83	555	n/a

Each return consisted of two components: numerical data for the year in question (quantitative data) and answers to a series of questions (qualitative data).

Every health board had a unique method for collecting, storing and analysing the returns sent to it. The health boards did not send any details to NHS Scotland (or other central body) but some produced regional reports that were shared with the PCTs in their area. Consequently it was not always possible to collect returns from each health board. Additionally, some health boards altered the reporting template to fit their needs, and not all health boards attempted to correct obvious errors in returns: meaning that we needed to make judgements about which returns to include (see Additional file 1).

### Quantitative analysis (2012-14)

Quantitative data consisted of:

- The practice population at the census date.
- Number of “significant event analyses” carried out during the 12 month period [13]
- Number “who died from cancer” during the 12 month period.
  - ○ Of these, the number who were on the palliative care register when they died and the number who had an electronic palliative care summary when they died.
- Number “who died from LTC other than cancer” during the 12 month period.
  - ○ Of these, the number who were on the palliative care register when they died and the number who had an electronic palliative care summary when they died.

**Fig. 1:**
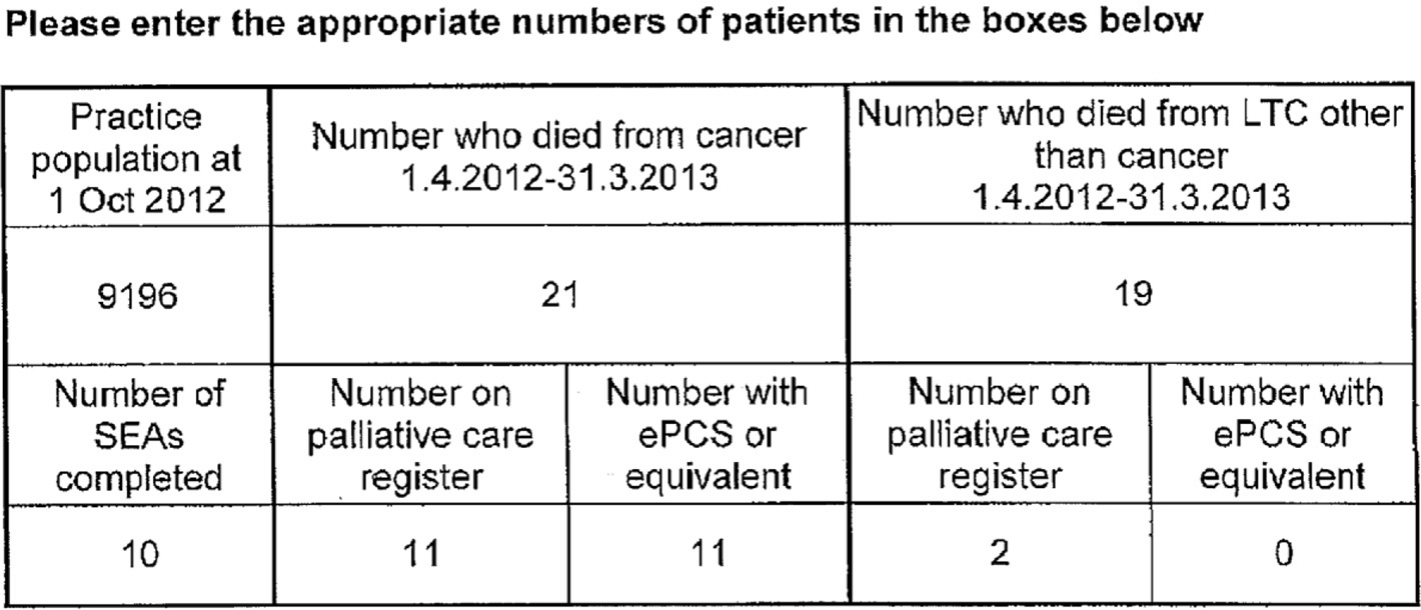
Example completed form showing quantitative data

An example of this data from one return is given in Fig. 1.

**Fig. 2:**
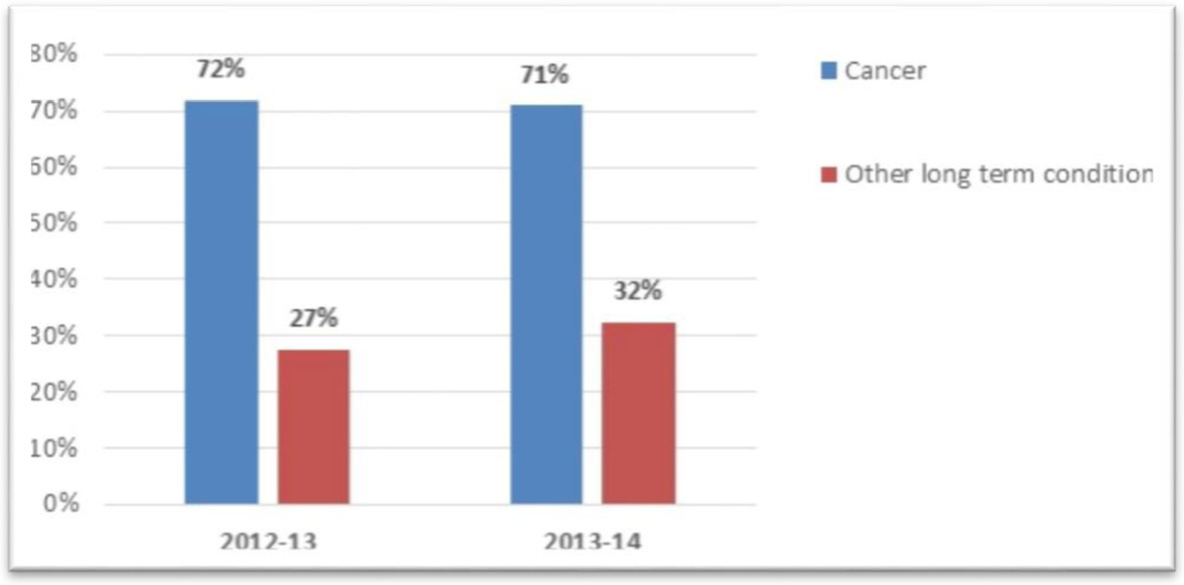
Percentage of deceased patients identified for palliative

In the returns we included for quantitative analysis (2012-13 *n* = 430, 2013-14 *n* = 555) there was a clear increase in the reporting of patients who “died from” a Long Term Condition (LTC) other than cancer while listed on the Palliative Care Register over the first two years of the DES. The incidence of 27 % in year one increased to 32.5 % in year 2. There was no discernible impact on the numbers who were reported as having died from cancer. (See Fig. 2 below).

In both years, a much greater proportion of patients who died from cancer were identified on practice palliative care registers prior to death, compared with those that were identified as dying from any other long term condition. For the 2012-13 data, there was a significant relationship between illness and whether or not the patient was identified on the palliative care register, *χ*^2^ (1) = 2822, *p* < .001, odds ratio = 6.76. The odds of a patient with cancer being identified on a palliative care register was over 6 times higher than the odds of a patient with a LTC being identified prior to death. The following year (2013-14) the odds of a patient with cancer being identified on a palliative care register was 5 times higher than the odds of a patient with a LTC being identified, *χ*^2^ (1) =2763, *p* < .001, odds ratio = 5.09, thus the imbalance was reduced but was a long way from being eliminated.

There was no association between year and identification on the PCR for patients with cancer (OR = 0.956, CI: 0.89-1.03), thus palliative care identification for patients with cancer remained stable from one year to the next. However, in 2013/14, the odds that patients with LTCs other than cancer were identified on the PCR were significantly higher compared with the previous year (OR = 1.27, CI: 1.19-1.35). This suggests a small but significant improvement in palliative care identification for this group of patients over time.

### Qualitative analysis (2012-13)

As part of the reporting process for the DES in both years in which we collected data, each GP practice (the “respondent”) was asked questions around identification of patients with palliative care needs, learning points for the practice from taking part in the DES, what their plans were to implement these learning points, and what could their regional health board do to better support the care of patients with palliative care needs. The responses were enumerated and categorised, and then an inductive thematic analysis was undertaken by BM to determine if there were common themes emerging. These themes were presented to a steering group for cross-checking. (See Additional file 2 for an example of a qualitative return).

- Practices sampled: 90
- Responses to identification questions: 90
- Learning points identified: 288
- Practice actions: 327
- Health board actions: 213

Three such themes emerged:

- Identification
- Communication & teamwork
- Management (workload and systems)

### Identification

An analysis of the qualitative data showed that respondents largely associated palliative care with terminal care: care provided in the last days and weeks of life. There was also a small minority who associated palliative care only with untreatable cancer. Because palliative care was associated with terminal care, a major barrier to initiating palliative care was the perceived difficulty of “breaking bad news” to a patient who might not be “ready” for such a conversation. The act of doing so was, therefore, seen as having a risk of causing harm. These difficulties were seen as greater in patients with LTCs other than those with cancer because they may not realise just how serious their illness is. Sample quotes are given below.

Because identification was seen as a necessary precursor to initiating palliative care and palliative care was seen as terminal care then GPs largely saw the issue of identification as one of prognosis: when could they accurately predict that a patient might be in their last few weeks of life? When asked whether they found identification of palliative care needs among patients with LTCs other than cancer *difficult or not*, approximately half of the practice respondents explicitly stated that they found it difficult (*n* = 44).

Of those who found it difficult to identify such patients, 19 explicitly linked the problem with the difficulty of determining when death was likely. Furthermore, of those who found it difficult, six indicated that they were reluctant to add LTC patients to the palliative care register (PCR) in case they stayed on longer than 12 months. In total 25 of the 44 who answered that identification was difficult associated identification directly or indirectly with prognosis rather than as a precursor to needs assessment.

#### Identification - quotes

Each quote is identified by a letter and number. The letter represents the health board the practice belongs to and the number represents which one of the practices in that health board is responding. To preserve confidentiality, there is no correlation between the code and the practice’s NHS number or Health Board name.

*We feel that we need to improve how we identify when patients with long term conditions (and particularly those with COPD) need to be placed on the palliative care pathway and also when they need to use an end of life pathway.* (F6)

*Can be difficult to predict the disease trajectory especially in non-malignant long term conditions - use of prediction tools can help to a certain level, but many factors influence decision as to when/if it is appropriate to use palliative care terminology.* (L11)

*The PHCT agreed that these patients could be difficult to identify and it would be difficult to know when to add them onto the palliative care list. This is due to the unpredictable cause and nature of some of the conditions, such as dementia or COPD, and also because some of our long term conditions were relapsing and remitting. It was also noted that with our large practice list size we did not wish to overwhelm the team with very large numbers of patients and that we should focus on those with the greatest need*. (T6)

### Communication and teamwork

Respondents identified the need to improve communication both with other professionals and with patients and families. Communication with patients and families was particularly difficult for the same reason that identification was: the association of palliative care with death and dying. Respondents were reluctant to identify patients because of difficulties in communicating this identification and were unable to communicate because they struggled to identify in the first place.

By far the greatest communication-based concern of respondents is communication with secondary care. Of the 42 responses around communication, 35 were focused on issues around secondary care. They expressed concerns around the following topics:

1. information lacking in discharge letters and speed of receipt of discharge letters;
2. difficulties in communicating with consultants while the patient is in hospital;
3. lack of communication about palliative care options by secondary care health teams with patients while the patient was in hospital.

*Communication from secondary care can be poor e.g. hand written discharge letters have limited information and often we don’t know what has been discussed with the patient or what the treatment plan is. Communication is often delayed. (A4)*

*Better communication from secondary care especially with regards to prognosis and terminal phase for cancer patients. (L1)*

*More timely information following discharge from secondary care. (L8)*

Related to communication, respondents strongly felt that improving their practice meetings was a fundamental step to providing better care. Of the 39 who responded in this way, 18 intended to expand a multi-disciplinary team meeting (MDTM) in some way, 16 intended to continue as at present, 5 were planning to review how it worked.

Various forms of expansion were planned:

- Increasing the range of participants involved in the MDTM
- Increasing the scope of patients and subjects to be covered
- Increasing the frequency of the meetings

*The practice will open up further discussion at multi-disciplinary meetings to include more LTC patients and if appropriate will add to the palliative care register. (A5)*

*A member of staff from nursing home will be invited to attend palliative care meeting. (K7)*

One barrier to improving MDTMs and communication in primary care was the (then) recent reorganisation of district nurse teams into geographical hubs, which had made provision of palliative care more difficult; GPs found it harder to make contact, arrange meetings and share information with the district nurse teams. There were also issues around under-resourcing of district nurse teams and IT access by district nurses.

*There is good communication within the Primary care setting though the change from District Nurses being placed in another Practice outwith our own has reduced the ease of communication. (L14)*

### Management

Respondents reported multiple practical barriers to improving identification and the management of patients with palliative care needs. Workload pressures were already great and adding more patients to a palliative care register was seen as increasing workload. Furthermore, the technology they were required to use: the electronic palliative care summary (EPCS) was time consuming and complicated to complete, required patient consent and lacked feedback about whether or not it had been successfully submitted. Consequently, respondents were reluctant to identify more patients if they had no way to manage the enlarged palliative care lists.

*I use ePCS regularly but have not found it user friendly and have tried to help many others get to grips with it – it is a pain linking the diagnosis, coding consent etc. It is also unclear which bits go through to OOH.* T1

*ePCS (VISION) is clumsy and complicated, only used by one GP for whole GP surgery.* A2

*I am unsure about the ePCS if at times the OOH doctors it is intended for have not been aware of it/able to access it. It would be good to know the technology is working in the way it is supposed to.* (L4)

Although the IT in place at the time was seen as a problem, many solutions proposed by respondents were focused on improving and extending the IT in order to help with managing patients.

*Improve information for OOH – Vision Summary and written. Training staff issue in practice to improve information being put in correct places in records.* K4

*Improving documentation in ePCS and formulation of anticipatory care plans.* K8

In addition, during the period under review, NHS Scotland was rolling out the Key Information Summary (KIS): an electronic anticipatory care record which was to replace the ePCS. Early adopters of the KIS were enthusiastic: finding it much easier to use and complete. Those who had not yet adopted it hoped it would solve many of the technological problems.

## Discussion

The quantitative data indicated that participants in the DES were more likely to identify patients for the palliative care register if they had cancer than if they had a non-malignant life-threatening condition (LTC). On the whole, participants reported that identification of patients with LTC was more difficult than those with cancer. Managing LTC was also more complicated due to the unpredictable trajectories of such conditions, however there are some indications that participation in the DES had improved their ability to identify patients with LTC.

The national scope of our findings strengthen previous research findings that GPs struggle most to identify patients with long-term conditions other than cancer for palliative care [4, 5, 14]. This project shows that there was a modest but significant increase in such identification during the period studied but we are unable to say what the cause of that increase is. This increase should indicate improved end-of-life care for patients with non-malignant long-term conditions across Scotland. It seems likely that further training and access to tools such as the SPICT™ [15, 16] or electronic record searching [17, 18] will further improve identification.

Research has shown that GPs tend to be reluctant to start palliative care at an earlier date than 2-3 months before an “expected” death and are reluctant to expand it to include patients with non-malignant long-term conditions [19]. Our research also illuminates the communicational, managerial, and technological issues facing the PCTs. Respondents saw the issues they faced as being grounded in poor information technology, complicated procedures, difficulties in communicating across the NHS and onerous book-keeping. Consequently their actions to improve their provision of palliative care often focused on improving their management and communication systems.

The introduction of the new electronic Key Information Summary (KIS) seems likely to significantly affect primary palliative care [20]. The KIS allows selected parts of the GP electronic patient record to be shared with the wider NHS using a template within the GP clinical system. Patients with the most complex health and social care needs are selected to have a KIS written to capture key points of their anticipatory care plans. It has a small extra section for palliative care which details any anticipatory prescribing and is designed to be added to as and when the patient’s clinical condition progresses. The incremental approach overcomes the barrier of requiring conversations that GPs see as risky before starting a KIS.

This evaluation also reveals the inherently multi-disciplinary nature of providing primary palliative care. Many of the problems raised by respondents came from the geographical reorganisation of district nurses that degraded many lines of communication. Likewise there were multiple issues around communicating with secondary care. The solutions proposed by respondents were grounded in improving ways of gathering multi-disciplinary teams for meetings and better coordination with consultants and specialist palliative care teams, including when the patient is in hospital. This is best understood as indicating the need for a whole team approach, where a patient does not “move” from one care team to another, but that secondary, primary and tertiary care are part of a holistic system [21, 22]. This is in keeping with the recent World Health Organisation resolution calling for the integration of palliative care in all settings, especially the community [23].

## Conclusion

Identification of patients with palliative care needs outside of cancer is still problematic. It is possible to increase the amount of such patients identified but that there remains a huge inequity.

Another difficulty in providing high quality palliative care lies in being able to communicate with other professionals in a timely manner. Making communication and information between sectors easier and more reliable would facilitate the multi-disciplinary and partnership working models that can be effective. Likewise, it appears that improvement of information technology, additional resources for training in its use and additional facilities such as computerising identification tools like the SPICT™ may make identification and management of patients easier and less time-consuming.

Our research shows “palliative care” with its connotations of imminent death and dying remains a barrier to PCTs undertaking actions associated with “palliative care”. This barrier in conjunction with the badly-designed IT systems for recording patients identified created a strong disincentive to PCTs to document the palliative care they provided during the initial period of the DES. The introduction of the Key Information Summary (KIS) throughout Scotland in 2013 appears to have improved the situation. The system is easier to use (though still requiring refinements) and allows GPs to undertake actions such as sharing information with Out Of Hours services without needing to frame those actions in terms of palliative care.

What we can learn two lessons from the DES in Scotland. Firstly, difficulties around identification of patients with non-malignant conditions for palliative care still remain, but can be addressed By recognising that early palliative care is a form of anticipatory care planning it appears that GPs feel enabled to act more promptly and communicate more effectively with patients. This should help remove some of the difficulties in professional-patient communication. Secondly, difficulties in communicating among professionals and working with poorly designed IT systems present practical issues to be overcome. Only through tackling both these issues are we likely to see more patients receive the type of early palliative care that could greatly improve their quality of life.

## Additional files

Additional file 1:
**Exclusion criteria for quantitative data.** (DOCX 15 kb) Additional file 2:
**Example qualitative return.** (DOCX 2516 kb)

## References

[1] Murtagh FE, Bausewein C, Verne J, Groeneveld EI, Kaloki YE, Higginson IJ (2014). How many people need palliative care? A study developing and comparing methods for population-based estimates. Palliat Med.

[2] Gómez-Batiste X, Martínez-Muñoz M, Blay C, Amblàs J, Vila L, Costa X (2013). Identifying patients with chronic conditions in need of palliative care in the general population: development of the NECPAL tool and preliminary prevalence rates in Catalonia. BMJ Support Palliat Care.

[3] Higginson IJ, Finlay IG, Goodwin DM, Hood K, Edwards AGK, Cook A (2003). Is there evidence that palliative care teams alter end-of-life experiences of patients and their caregivers?. J Pain Symptom Manage.

[4] Harrison N, Cavers D, Campbell C, Murray SA (2012). Are UK primary care teams formally identifying patients for palliative care before they die?. Br J Gen Pract.

[5] Zheng L, Finucane AM, Oxenham D, McLoughlin P, McCutcheon H, Murray SA (2013). How good is primary care at identifying patients who need palliative care? A mixed methods study. Eur J Palliat Care.

[6] Addington-Hall JM, Hunt K, Cohen J, Deliens L (2012). Non-cancer patients as an under-served group. A Public Health Perspective on End of Life Care.

[7] Living and Dying Well: a national action plan for palliative and end of life care in Scotland [http://www.scotland.gov.uk/Publications/2008/10/01091608/0]. Accessed 23/11/2015.

[8] Scottish Government (2011). Living and Dying Well: Building on Progress.

[9] Government S. The primary medical services directed enhanced services (Scotland) 2012 palliative care In*.* Edited by Directorate HaSCI. Edinburgh: NHS Scotland; 2012.

[10] Hall S, Murchie P, Campbell C, Murray SA. Introducing an electronic Palliative Care Summary (ePCS) in Scotland: patient, carer and professional perspectives. Fam Pract. 2012;29(5):576-85. 10.1093/fampra/cms011 22337868

[11] Addington-Hall JM, Bruera E, Higginson IJ, Payne S, Payne S (2007). Qualitative methods of data collection and analysis. Research methods in palliative care.

[12] Pope C, Ziebland S, Mays N (2000). Analysing qualitative data. BMJ.

[13] de Wet C, Bradley N, Bowie P (2011). Significant event analysis: a comparative study of knowledge, process and attitudes in primary care. J Eval Clin Pract.

[14] Murray SA, Boyd K (2011). Using the ‘surprise question’ can identify people with advanced heart failure and COPD who would benefit from a palliative care approach. Palliat Med.

[15] Highet G, Crawford D, Murray SA, Boyd K (2014). Development and evaluation of the Supportive and Palliative Care Indicators Tool (SPICT): a mixed-methods study. BMJ Support Pall Care.

[16] Maas EAT, Murray SA, Engels Y, Campbell C. What tools are available to identify patients with palliative care needs in primary care: a systematic literature review and survey of European practice. BMJ Support Pall Care. 2013;3(4):444-451. 10.1136/bmjspcare-2013-000527 24950525

[17] Mason B, Boyd K, Murray SA, Steyn J, Cormie P, Kendall M (2015). Developing a computerised search to help UK General Practices identify more patients for palliative care planning: a feasibility study. BMC Fam Pract.

[18] Thomas K. Finding patients who may die: Electronic searching for people with palliative care needs. In: International Congress on Palliative Care. Montreal, Canada: Montreal McGill University; 2012.

[19] Boyd K, Mason B, Kendall M, Barclay S, Chinn D, Thomas K (2010). Advance care planning for cancer patients in primary care: a feasibility study. Br J Gen Pract.

[20] Craig J, Morris L, Cameron J, Setters J, Varley D, Lay A (2015). An evaluation of the impact of the key information summary on GPs and out-of-hours clinicians in NHS Scotland. Scott Med J.

[21] Daveson BA, Harding R, Shipman C, Mason BL, Epiphaniou E, Higginson IJ (2014). The real-world problem of care coordination: a longitudinal qualitative study with patients living with advanced progressive illness and their unpaid caregivers. PLoS One.

[22] Mason B, Epiphaniou E, Nanton V, Donaldson A, Shipman C, Daveson B (2013). Coordination of care for individuals with advanced progressive conditions: a multi-site ethnographic and serial interview study. Br J Gen Pract.

[23] Assembly WH. Strengthening of palliative care as a component of integrated treatment within the continuum of care. In*.*, vol. EB134/SR/8. Geneva, Switzerland: World Health Organisation; 2014.

