# Supplementary material for: Improving primary palliative care in Scotland: lessons from a mixed methods study
Source: BMC Fam Pract. 2015 Dec 10;16:176. doi: 10.1186/s12875-015-0391-x (PMC4676155; doi:10.1186/s12875-015-0391-x)
Supplement: Additional file 1: — Exclusion criteria for quantitative data. (DOCX 15 kb) [file 12875_2015_391_MOESM1_ESM.docx]

# Appendix 1 Exclusion criteria for quantitative data

Misinterpretation of the reporting template was evident. Several types of misinterpretation occurred:

- Several practices reported more people having died after being added to the Palliative Care Register than had died overall during the report period.
- In a number of practices, more people were reported as having an epcs or equivalent, than had actually died of cancer or a long term condition other than cancer.
- Some practices provided a breakdown of those on the palliative care register for those on whom an SEA was carried out as opposed to all patients who died with cancer or a long term condition.
- Other errors included: reporting total patient deaths including sudden deaths; reporting only deaths of those on the PCR as opposed to all deaths.

**Table 5: Common misinterpretations in relation to DES Level 2 report completion 2012-13**

| **Des template completions - Misinterpretation type** | **No. of practices** | **% of practices** |
| --- | --- | --- |
| 1: Reported greater number of patients with cancer on the palliative care register that had died from cancer | 43 | 8.4% |
| 2: Reported greater number of patients with long term condition on the palliative care register than had died from long term condition other than cancer | 20 | 4.0% |
| 3: Reported greater number of patients with cancer with an EPCS than had died from cancer. | 25 | 5.0% |
| 4: Reported greater number of patients with LTC with an EPCS than had died from LTC | 10 | 2.0% |
| 5: No. of SEAs were equal to the total number of patients on the palliative care register. | 31 | 6.0% |
| 1. 6. Other misinterpretations | 13 | 2.5% |
| **Any of the above** | **82** | **16%** |
