# Supplementary material for: Improving primary palliative care in Scotland: lessons from a mixed methods study
Source: BMC Fam Pract. 2015 Dec 10;16:176. doi: 10.1186/s12875-015-0391-x (PMC4676155; doi:10.1186/s12875-015-0391-x)
Supplement: Additional file 2: — Example qualitative return. (DOCX 2516 kb) [file 12875_2015_391_MOESM2_ESM.docx]

# Appendix 2 – example qualitative return

**Palliative Care: Reporting Template 2012-13 for level 2 activity**

**PALLIATIVE CARE DES LEVEL 2 ACTIVITY – PRACTICE REPORT**

**Practice Name and Code: [[[removed]]]**

| 1. **Examining the care of the patients who died, under the following headings, are there emerging themes of note under any of the headings**  - **Identification of patients with non-malignant illness who have palliative care needs?**   ***Do you find this difficult? Did the guidance in Annex B or SPICT help?***  It is difficult to identify such patients. Although the guidance was felt to be some help it was felt to be too general. It was accepted however that it would be difficult to make it more specific   - **Expected or unexpected death**   ***If any of your patients who died from a LTC in the past year were not on your palliative care register then***   - ***consider the reasons for this*** - ***Could it have been predicted by using the guidance in Annex B or SPICT?***   The SEAs looked at 2 cancer patients who were not on the palliative care register. Both of these patients were diagnosed in hospital and died in hospital quickly from their illness. There were no specific practice learning points identified for change in either case.  There were 2 SEAs looking at non cancer patients. One of these patients was in a long term care home and it was recognised that it can be difficult with such patients to identify when they are become end of life. Usually the family is not present at the time they are seen and as their social needs are addressed in the home this doesn’t lead to a specific discussion other than dealing with specific clinical issue that day. An increased awareness of this from the doctors having discussed the SEA is helpful with this. It was also recognised that with eKIS and anticipatory care planning that patients such as this in long term care could be looked at to ensure that patients and families wishes were recorded so the were known to the out of hours service.  The other SEA of a patient who died of non cancer long term condition had a death at home well supported by her family. She had a long illness and had essentially been looked after very well for a number of years by her very caring family. She had one out of hours contact in June 2011 and all other care was by the family and the practice. Although it was agreed she would have been suitable for the palliative care register there was no change in outcome as a result her not being on it.   - **ePCS or equivalent**   ***If any of your patients who died from a LTC in the past year did not have an ePCS then***   - ***consider the reasons for this*** - ***Would it have been helpful?***   All patients on the register had an ePCS completed. The patients who were not on the palliative care register had no change to their outcome by not having one. It was recognised with one of the non cancer SEA patients at discussion that if they had been added earlier to the palliative care register that it would have been more helpful. This can be difficult in patients in long term establishments who are often seeing a different doctor for their house visit. If the same Dr was seeing the patient the pattern of deterioration may be identified earlier but it was agreed such patients in long term care placements would benefit with the introduction eKIS and anticipatory care planning.   - **Do Not Attempt Cardiopulmonary Resuscitation (DNA CPR) status**   ***If any of your patients who died from a LTC in the past year did not have DNACPR discussed then***   - ***consider the reasons for this*** - ***Would it have been helpful?***   DNACPR was present in 50% of the last 10 deaths on the palliative care register and on 80% of those who had died at home. In the current register it was present in 2 out of 16 of the patients. The main difficulty was when it was felt appropriate to ask this question and how often the ePCS was updated. There was no death that didn’t have a DNACPR form where it would have been helpful.     - **Preferred place of care/death**   ***How often was this discussed and if so were the patients wishes honoured?***  All patients were discussed at least monthly at the palliative care meeting with those with more active needs discussed weekly.  21 out of 24 patients on the palliative care register had a preferred place of death recorded. – 87.5%  Of the 21 recorded 14 died where the patient had wished. – 66.67%   - **Use of end of life care pathways**   ***If any of your patients who died from a LTC at home or in a care home in the past year were not on an end of life care pathway then***   - ***consider the reasons for this*** - ***Would it have been helpful?***   The care of the dying pathway is usually completed by the district nursing team looking after the patient rather than the medical staff. The district nursing team had all patients who died at home of a long term condition on a care of the dying pathway. The patients who were not on the palliative care register were not on a care of the dying pathway from the district nursing team but this had no effect on their death. |
| --- |
| 1. **List 3 (or more) Learning Points for the Practice as a whole or individuals**  - Develop better communication with the hospice nurses - Develop system where hospice fax change of medication requests - Consider palliative care register addition as part of dementia review process - Development of non palliative care plans in long term care patients - Our local xray facility helped with speed of diagnosis |
| 1. **What does the Practice intend to do to implement these learning points?**  - Plan a meeting to meet the hospice nurses at one of our practice lunchtime meetings to discuss better ways to communicate generally and look at system for change of medication - Dr [[[name removed]]] the QOF lead on dementia will look at the dementia review protocol re including a palliative care consideration. - For the practice through the CHP to continue to support the value of a local xray facility - With the introduction of anticipatory care planning this should help with looking earlier at the wishes of patients and their families in long term care |
| 1. **What needs to happen within your NHS Board area to support and improve the care of patients with palliative care needs? E.g. communication with secondary care, additional access to other services, guidelines, training, IT etc.**   Better sharing of information from secondary care – often we don’t get correspondence for weeks after patients have been seen.  More time (resource) to spend with these patients. Our time is being more and more squeezed by new contract demands which do not come with a new resource to help to achieve them. Good palliative care takes considerable time.  Recognition of the benefit of the local xray facility and support to keep the local service. |
